# Allostatic Load Following Short-Term Intervention: Cognition in Older Hypertensive Adults

**DOI:** 10.1093/geroni/igab046.3664

**Published:** 2021-12-17

**Authors:** Anthony Cirilo, Jordan Kohn, Gavrila Ang, Meredith Pung, Emily Troyer, Christopher Pruitt, Laura Redwine, Suzi Hong

**Affiliations:** 1 UCSD MADURA R25 Advancing Diversity in Aging Research Program, San Diego, California, United States; 2 University of California, San Diego, San Diego, California, United States; 3 University of South Florida, Tampa, Florida, United States

## Abstract

Allostatic load (AL), a measure of cumulative effect of prolonged stressors across physiological systems, is consistently associated with adverse health outcomes. Greater AL is correlated with functional decline in aging, but effects of behavioral interventions, such as Tai Chi (TC), on AL in older adults in a short-term is unknown. To investigate the effects of TC practice on AL and cognitive function and an AL-cognition relationship, older adults (60-95 years) with hypertension were recruited and randomly assigned to 12-week TC or Healthy Aging Practice-centered Education (HAP-E) classes. The AL index (ALI) included: SBP and DBP; urinary epinephrine and norepinephrine; plasma inflammatory biomarkers (CRP, IL-6); metabolic biomarkers (HDL, total cholesterol, triglycerides, HbA1c); and BMI. The Montreal Cognitive Assessment (MoCA) was administered to assess cognitive function. Generalized linear mixed-effects models, adjusted for age, race, education, and intervention attendance, was used. Pre- and post-intervention ALI did not change significantly in TC (2.61 (1.48) to 2.76 (1.62)) or HAP-E (2.84 (1.61) to 2.66 (1.86)). High ALI was associated with lower MoCA scores, indicating poorer cognitive performance (IRR=0.96; 95% CI: 0.93-0.98; p=0.002) across the time points. Of note, the MoCA scores did not significantly change across time (25.4 (3.2) to 26.0 (3.0)). 12-week TC or HAP-E interventions did not lead to a significant change in ALI or cognitive performance in our population. However, our findings show greater AL theoretically attributed to chronic stress is associated with cognitive functioning in older adults consistently over about 4 months.

